# ConSurf‐DB: An accessible repository for the evolutionary conservation patterns of the majority of PDB proteins

**DOI:** 10.1002/pro.3779

**Published:** 2019-11-22

**Authors:** Adi Ben Chorin, Gal Masrati, Amit Kessel, Aya Narunsky, Josef Sprinzak, Shlomtzion Lahav, Haim Ashkenazy, Nir Ben‐Tal

**Affiliations:** ^1^ Department of Biochemistry and Molecular Biology, George S. Wise Faculty of Life Sciences Tel Aviv University Tel Aviv Israel; ^2^ School of Molecular Cell Biology & Biotechnology, George S. Wise Faculty of Life Sciences Tel Aviv University Tel Aviv Israel

**Keywords:** binding site, ConSurf, ConSurf‐DB, evolutionary conservation, evolutionary rate, functional importance

## Abstract

Patterns observed by examining the evolutionary relationships among proteins of common origin can reveal the structural and functional importance of specific residue positions. In particular, amino acids that are highly conserved (i.e., their positions evolve at a slower rate than other positions) are particularly likely to be of biological importance, for example, for ligand binding. ConSurf is a bioinformatics tool for accurately estimating the evolutionary rate of each position in a protein family. Here we introduce a new release of ConSurf‐DB, a database of precalculated ConSurf evolutionary conservation profiles for proteins of known structure. ConSurf‐DB provides high‐accuracy estimates of the evolutionary rates of the amino acids in each protein. A reliable estimate of a query protein's evolutionary rates depends on having a sufficiently large number of effective homologues (i.e., nonredundant yet sufficiently similar). With current sequence data, ConSurf‐DB covers 82% of the PDB proteins. It will be updated on a regular basis to ensure that coverage remains high—and that it might even increase. Much effort was dedicated to improving the user experience. The repository is available at https://consurfdb.tau.ac.il/.

**Broader audience:**

By comparing a protein to other proteins of similar origin, it is possible to determine the extent to which each amino acid position in the protein evolved slowly or rapidly. A protein's evolutionary profile can provide valuable insights: For example, amino acid positions that are highly conserved (i.e., evolved slowly) are particularly likely to be of structural and/or functional importance, for example, for ligand binding and catalysis. We introduce here a new and improved version of ConSurf‐DB, a continually updated database that provides precalculated evolutionary profiles of proteins with known structure.

## 1 INTRODUCTION

The explosion of protein sequence data over recent decades has led to the emergence of numerous databases that organize and characterize protein sequences according to biologically relevant features. These databases enable researchers to extract invaluable information on many proteins quickly and inexpensively. About 12 years ago, our group introduced ConSurf‐DB, a database aimed at providing researchers with convenient access to evolutionary data for proteins of known structure.^1^ Herein, we present a new version of ConSurf‐DB.

In general, evolutionary information serves as a powerful tool in studies of protein structure and function, and it is especially useful for identifying residues with important functional roles. In particular, residues that are involved in functions such as ligand binding and catalysis, or that are necessary for maintaining the protein's structure, tend to be evolutionarily conserved, meaning that during protein evolution their positions tend to change more slowly than other positions.^2^ This tendency results from the fact that mutations to functionally important residues may compromise the protein's function and/or structural stability and as such are unlikely to be tolerated. Moreover, once the evolutionary rates of a protein's amino acid positions have been calculated, it can be highly informative to map these rates onto the protein's three‐dimensional structure: By observing where conserved residues are located within the protein's structure, researchers may be able to predict what the functional roles of these residues might be. Evolutionary information can also guide experimental effort, such as mutagenesis, to confirm such predictions and to decipher the protein's mechanism of action.

The extraction of evolutionary data for a given query protein is based on the comparison of that protein to its homologues, that is, other proteins of a shared evolutionary origin. There are several different approaches and methods that infer evolutionary information from homologues.^3^ These are all based on aligning the query and its homologues to each other in a way that maximizes the total similarity in all the amino acid positions, that is, a multiple sequence alignment (MSA). The simplest estimates are based on the consensus approach: For each position, the amino acid that appears in that position in the greatest number of homologues is identified; then, the evolutionary conservation level of that position is determined according to whether the “frequency” of the amino acid (i.e., the proportion of homologues in which it appears) exceeds a predefined consensus threshold. More sophisticated methods estimate conservation using the entropy of each position, calculated from the collective frequencies of the different amino acids that appear in that position.^4^, ^5^ These approaches are highly sensitive to the specific selection of homologues used because they do not account for the phylogenetic relationships among homologues. Thus, results obtained using very close homologues may differ substantially from calculations with a more diverse set. To alleviate this problem, tools such as the Evolutionary Trace Viewer^6^ and SiteFiNDER|3D^7^ use phylogenetic trees, which reflect the evolutionary relationships between the proteins. Explicit consideration of the evolutionary relationships among the homologues helps to reduce inaccuracies caused by uneven sampling in sequence space and decreases the sensitivity to the choice of homologues. Notably, whereas Evolutionary Trace Viewer and SiteFiNDER|3D are based only on sequence information, an alternative tool, FuncPatch,^8^, ^9^ also accounts for the three‐dimensional structure of the protein. This approach is based on a phylogenetic Gaussian process that accounts for three‐dimensional correlation of substitution rates in different positions according to the tertiary structure of the protein.

The most commonly used tool for calculating evolutionary rates on the basis of sequence information, while accounting for the phylogenetic tree, is ConSurf.^10^, ^11^ In ConSurf, homologues of the query sequence are detected and aligned, a phylogenetic tree is constructed, and the evolutionary rates of all positions in the query protein are then calculated using the Rate4Site program^12^ without explicit use of the three‐dimensional structure of the protein. Specifically, Rate4Site estimates the evolutionary rates of the amino acids, by taking into account the relationships among the homologues and the evolutionary process, as reflected in the phylogenetic tree. Rate4Site also assigns a credibility interval for the evolutionary rates. The conservation grades (derived from the evolutionary rates) are projected onto the corresponding positions in the query sequence, where each position is colored according to a unique color‐coding scale ranging from least to most conserved. ConSurf also maps the conservation grades onto the three‐dimensional structure of the protein, if available. This step enables the evolutionary information to be integrated with spatial considerations that are visible only from the structure, for example, the location of binding/catalytic sites and ligand‐binding positions.

Though ConSurf carries out its calculations relatively quickly, in certain cases (e.g., high‐throughput studies involving many proteins) scholars may prefer to get an instant conservation map of a protein's structure, without having to enter specific calculation parameters. ConSurf‐DB was introduced to address these cases.

ConSurf‐DB is a repository of precalculated evolutionary rates for the protein structures deposited in the Protein Data Bank (PDB),^13^, ^14^ the main resource for experimentally determined protein structures. The PDB is constantly growing; it currently contains nearly 150,000 entries representing protein structures (according to http://www.rcsb.org/), three times more than it did 12 years ago, when ConSurf‐DB was introduced. To accommodate this growth and to adapt the database to recent methodological developments, we have designed a new release of ConSurf‐DB. The current version of the database covers 82% of the PDB and will be periodically updated to include new PDB entries, as well as to exploit the flood of sequence data. ConSurf‐DB is available as an online website and does not require local installation.

## 2 METHODOLOGY FOR CREATION OF THE CONSURF‐DB REPOSITORY

**Figure 1:**
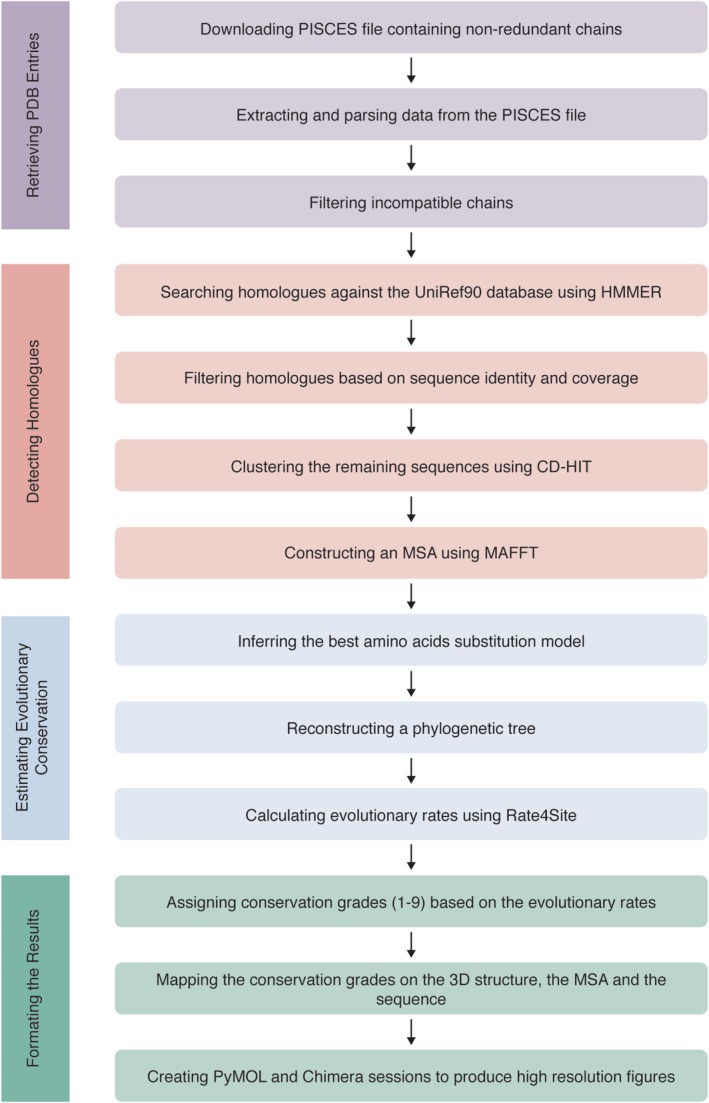
A flowchart of the pipeline used to construct ConSurf‐DB. The pipeline consists of four steps: retrieving PDB entries, homologue detection and building a multiple sequence alignment, estimating evolutionary conservation, and formatting the results

The new version of ConSurf‐DB is based on a fully automated process that consists of four main steps: downloading and parsing nonredundant PDB entries, collecting sequence homologues and aligning the sequences, calculating evolutionary rates, and finally formatting the results for presentation in the ConSurf‐DB website (Figure 1). Separation of these individual steps provides flexibility and modularity, enabling new data—for example, updates to the PDB—and new features to be integrated efficiently. The repository will be updated frequently, where each update involves making calculations for newly added PDB entries, as well as revisiting old PDB entries that were not eligible for inclusion in previous compilations (e.g., because of an insufficient number of homologues). Once a year, the whole database will be reconstructed for the entire PDB, in order to account for new homologues that have become available as a result of growth in sequence data.

The first step in building ConSurf‐DB is retrieving the PDB entries. Each PDB entry can contain one or more protein chains, which are handled separately in ConSurf‐DB. In order to overcome the problem of redundancy in the PDB (i.e., more than one structure for a given protein sequence), the chains are extracted from a PISCES file (downloaded from http://dunbrack.fccc.edu/).^15^, ^16^ This file contains all nonredundant (unique) chains in FASTA format, where the header of each unique chain lists all redundant chains, that is, chains with 100% sequence identity. After extraction of the unique chains, their sequences, and their identical chains from the file, the unique chains are filtered using the following criteria: “length”, “PDB file” and “modifications”. The “length” filter eliminates chains containing fewer than 30 residues, as for shorter chains it can be challenging to collect credible homologues and construct a reliable phylogenetic tree. The “PDB file” filter discards chains that do not have a PDB file, either because the entry has become obsolete or because they are too large (containing 100,000 atoms or more). Large structures are deposited in the PDB only using the mmCIF format, which the ConSurf‐DB pipeline cannot handle yet (though it soon will). Finally, the “modifications” filter handles the chains that contain nonstandard amino acids. Each such amino acid is modified to its closest neighbor among the standard amino acids, and if the fraction of these modified residues in the chain exceeds 15%, the chain is filtered out. In any case, the modifications are saved to the chain's data. Following this initial filtration, a directory containing the input data is constructed in the repository for each of the remaining unique chains, and they are associated with their sequences and identical chains. Thus, each unique chain's calculations can easily be mapped to the structures of all its identical chains.

The second step is searching for sequence homologues in UniRef90,^17^, ^18^ a clustered version of the UniProt database.^19^, ^20^ This is done using one iteration of the homologue search tool HMMER^21^, ^22^ with an *E*‐value threshold of 0.0001. The candidate homologues retrieved by HMMER for a certain chain are further filtered according to the following three parameters: (a) sequence identity—first, sequences identical to the query by over 95% are discarded to reduce error due to sample bias; (b) sequence coverage—sequence homologues that cover below 60% of the query protein are filtered; and (c) maximum overlap among homologues—some homologous sequences may overlap. In this case, if the overlap is greater than 10%, the highest scoring homologue is chosen, and the others are discarded. After this filtration process, chains with fewer than 50 homologues are eliminated. In ConSurf, the minimum number of homologues required to calculate evolutionary rates is five; here, we adopt a higher threshold with the aim of ensuring that the estimated evolutionary rates included in ConSurf‐DB are more robust. Next, cluster database at high identity with tolerance (CD‐HIT)^23^, ^24^ removes any redundant homologues with a threshold of 95%. If there are more than 50 homologues after the CD‐HIT filtration process, the remaining homologues are sorted by their *E* value in ascending order, in line with the principle that the lower the *E* value the more significant the resemblance between the homologue and the query protein. A maximum of 300 homologues are sampled uniformly from the sorted list to create the final list of homologues of the query protein. This is also a higher threshold in comparison to the default threshold used in ConSurf (150 homologues); again, the aim is to increase the robustness of the results. Finally, an MSA of the homologues is constructed using the MAFFT‐LINSi procedure.^25^, ^26^

The third step is estimating the evolutionary rate at each amino acid position. To this end, the MSA is first used to infer the best amino acid substitution model.^27^ This model essentially describes the evolution of the amino acids. Several such models are considered, including the following: JTT,^28^ LG,^29^ Dayhoff,^30^ WAG,^31^ mtREV,^32^ and cpREV.^33^ Next, a phylogenetic tree is built from the MSA with the Neighbor‐Joining method,^34^ implemented in Rate4Site. Finally, Rate4Site assigns an evolutionary rate to each position in the query sequence, based on the phylogenetic tree and the substitution model, and using an empirical Bayesian methodology.^35^ The evolutionary rates are normalized around zero, where rapidly evolving (variable) positions are assigned positive values and slowly evolving (conserved) positions are assigned negative values. In addition, a confidence interval, estimated using the empirical Bayesian method,^36^ which represents the extent of credibility of the estimated evolutionary rate, is assigned to each position. Finally, the evolutionary rates are categorized into discrete conservation grades, ranging from 1 to 9, where 1 represents the most highly variable residue positions, 5 represents positions of intermediate conservation, and 9 represents the most highly conserved positions. These grades are then mapped to nine colors, providing a clear and intuitive means of visualizing the conserved and variable regions in the protein. Positions that are assigned grades with low confidence are treated as a separate, tenth, category.

The final step is formatting and visually representing the data, to make the information accessible and user friendly. The conservation grades (colors) are mapped onto the three‐dimensional structure of the query protein, which can be viewed using the NGL viewer^37^, ^38^ or FirstGlance in Jmol.^39^ This visualization is highly enlightening because it emphasizes the important, evolutionarily conserved regions of the protein. The colors are also projected on the query sequence and on the MSA. Moreover, session files presenting the protein structure, colored according to the conservation grades, are created using the PyMOL^40^ and UCSF Chimera^41^ programs. All visual results are available in two color scales: the default color scale, which is cyan‐through‐maroon and the color‐blind friendly color scale, which is green‐through‐purple. These color scales correspond to variable (Grade 1)‐through‐conserved (Grade 9). Positions with low reliability according to the confidence interval are colored in light yellow in both color scales. Additional nonvisual data are also available to users, as well as links to related sources of information such as PDBsum^42^, ^43^ and Proteopedia.^44^, ^45^ The repository can be accessed through a website, available at https://consurfdb.tau.ac.il/. To view the results, users need only to provide the PDB ID or sequence of the query protein.

## 3 NEW FEATURES

### 3.1 Homologue detection using HMMER

In previous releases of ConSurf‐DB, the homologues of the query protein were collected using PSI‐BLAST. Yet, new sequence search methodologies have developed in recent years, to keep pace with the continuous increase in the number of protein sequences. In the new release of ConSurf‐DB (as well as in ConSurf itself), homologues are collected using the more advanced HMMER algorithm. HMMER implements probabilistic inference using profile hidden Markov models. Given a query sequence *x* and a target sequence *y*, BLAST calculates the score of the optimal alignment of *x* and *y*, whereas HMMER calculates a score that is the sum of scores of all possible alignments of *x* and *y*. Because HMMER uses a heuristic acceleration algorithm, it remains similar in speed to BLAST, but with a better rate of correctly detected homologues and a much lower rate of falsely detected hits. Implementation of HMMER in the new release of ConSurf‐DB has improved homologue identification.

### 3.2 Batch download

Since results are precalculated in ConSurf‐DB, we can provide results for several protein structures in a single download. This feature, which was not included in previous versions of ConSurf‐DB, is now available on our homepage, and users can access it by uploading a list of desired chains (where each chain appears on a new line).

### 3.3 Improved visualization

- Improving the color scales. In this release of ConSurf‐DB, the colors, both in the default and color‐bind scales, were refined to allow better distinction between the different conservation grades.
- Providing PyMOL session files for high‐resolution figures. PyMOL is a popular molecular visualization program; it contains various functions that enable users to analyze three‐dimensional structures of proteins (e.g., show hydrogen bonds, calculate electrostatic potential), and it can also be used to create high‐resolution images of the viewed protein. In previous versions of ConSurf and ConSurf‐DB, users were provided with a modified PDB file of their protein, which contained the conservation grades in the temperature factor column. Using this file and a provided script, users were able to color the protein according to its calculated conservation grades. In this version, we provide a complete PyMOL session file, in which the query protein is already colored according to conservation. To create a high‐resolution image, the user needs only to open the file with PyMOL and save it as a figure. While working on this feature, we discovered and fixed some issues with the coloring script. We therefore recommend that users who prefer to construct their own ConSurf figures download the revised files provided in this version.
- Color‐blind presentation option for all visual results. In earlier releases of ConSurf‐DB, the visual results were presented using only the default conservation color scale. From this version on, all visual results will be available in both the default and the color‐blind scales, both for viewing directly and for downloading. The color‐blind display can be selected in the homepage, when running a query, or alternatively, in the results page, by clicking a button that enables switching between the two displays.
- Supporting the NGL viewer. The page of each entry now includes a visualization of the three‐dimensional structure using the NGL viewer. This viewer is very fast and provides many features, such as zooming in on the interactions of the query protein with its cognate ligand, thus highlighting important biological information.

### 3.4 Improvements in design and user experience

The new release of ConSurf‐DB is considerably more user friendly than the previous release and includes many improvements in the user interface and user experience. In terms of the query process, for example, the list of protein chains is presented in a drop‐down menu in the homepage, instead of on a new page. In terms of technical support, a contact form is now available to improve our communication with users. We encourage our users to write, and we would appreciate any feedback.

Moreover, in this version of ConSurf‐DB, we present a new design for the website, which should improve clarity of presentation and ease of use. For example, in the new results page, the order of the results is determined by anticipated importance and usefulness, making it easier for users to find what they need. In addition, the names of the result files are much more intuitive and informative, and users can further access a README file that provides detailed information for all results. Finally, the running parameters of ConSurf‐DB are presented in the results page, for the user's convenience.

## 4 CONSURF‐DB IN NUMBERS

The statistics for this version of ConSurf‐DB are presented in Table 1. ConSurf‐DB was built on the basis of a PISCES file containing 108,958 nonredundant protein chains from the PDB (at 100% sequence identity threshold); the PISCES file was updated on September 2019. Of this initial set, we filtered 7,054 chains shorter than 30 amino acids, 4,629 chains from large structures, and 210 chains with more than 15% modified amino acids, which, as explained above, are not suitable for the calculation. A total of 97,065 nonredundant chains remained after this initial filtration. The homologue search for each of these chains was performed using HMMER v3.2.1 against UniProt/UniRef90 release 07‐2019. The homologues were filtered by thresholds and using CD‐HIT v4.7 and were then aligned using MAFFT v7.419. The build process was carried out using 150–200 CPUs, with an average CPU time of roughly 15 min per chain. For 7,363 of the 97,065 chains, we failed to find at least 50 homologues and aborted calculation.

**Table 1:** Statistics of ConSurf‐DB

PDB chains		MSA sizes	
Total chains found	473,197	Chains with less than 50 homologues	7,363
Total nonredundant chains found	108,958	MSA's created	
Filtered		Chains with 50–100 homologues	3,238
Chains shorter than 30 amino acids	7,054	Chains with 101–200 homologues	4,978
Chains with large structures	4,629	Chains with 201–300 homologues	81,486
Chains with more than 15% modified residues	210	Total chains processed	89,702
Total chains post‐initial filtration	389,863		
Total nonredundant chains post‐initial filtration	97,065		

*Note*: Currently, the databases cover 89,702 of the 108,958 protein chains in the nonredundant set, that is, 82%.

In aggregate, as of November 2019, ConSurf‐DB covers 89,702 of the 108,958 unique protein chains in the PDB, that is, coverage of 82%, corresponding to a total of 365,218 chains. The vast majority of the calculations are based on large MSAs of 201–300 homologous proteins.

## 5 EXAMPLES OF APPLICATIONS OF EVOLUTIONARY DATA: ACTIVE SITE ANALYSIS IN ENZYMES AND ANTIBODIES

As discussed above, data regarding the degree of conservation of each position in a protein can be used to predict the biological significance of specific positions, as functionally important positions tend to be more evolutionarily conserved compared with other positions. The high conservation of functional positions results from negative selection on mutations in these positions, as such mutations may result in loss of function.

**Figure 2:**
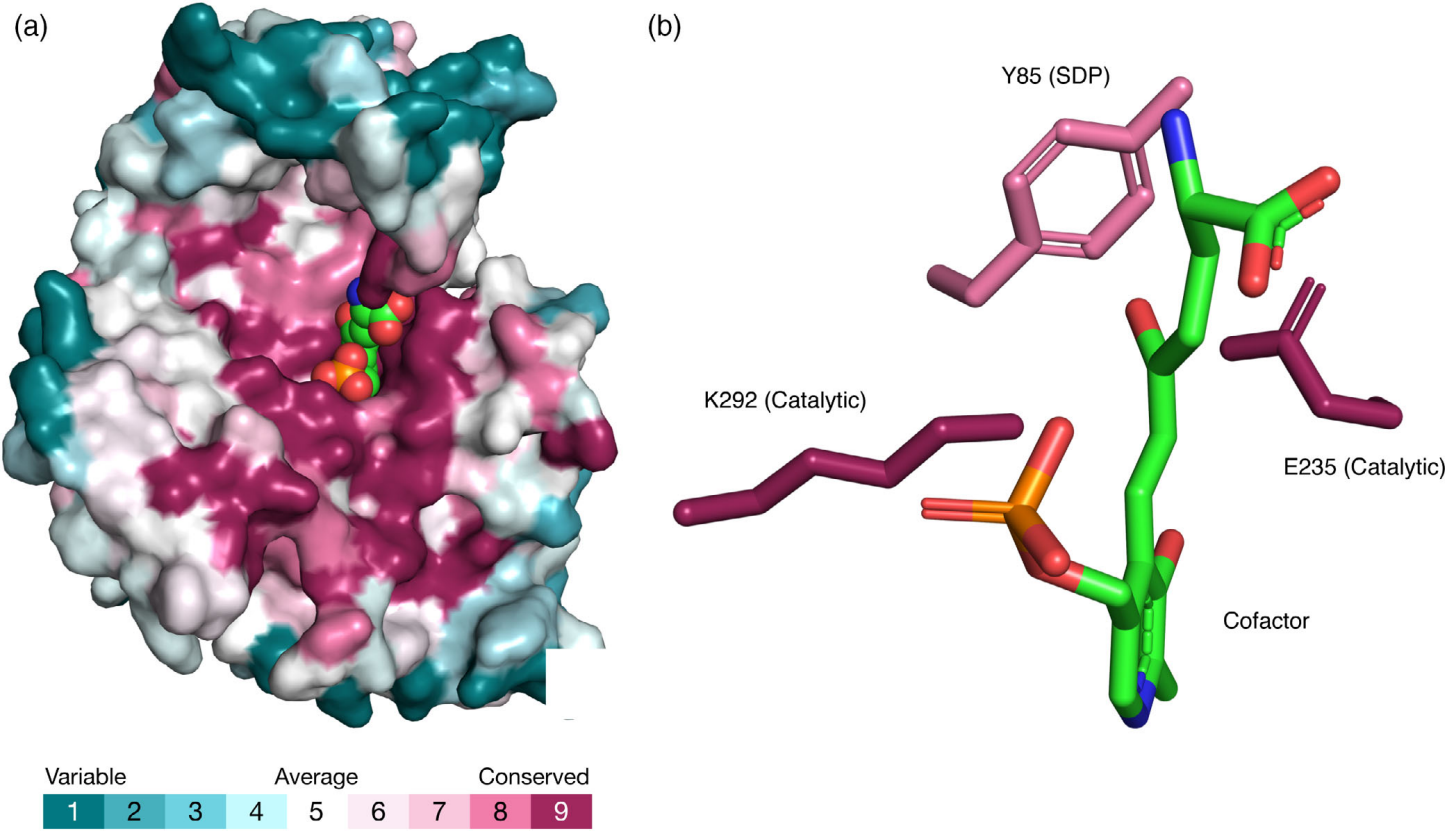
Conservation of catalytic and specificity‐determining positions (SDPs) in the active site of Or‐AT (PDB entry 2oat). (a) Ornithine‐aminotransferase, colored by conservation grade and shown in surface representation, together with the inhibitor–cofactor (pyridoxal phosphate) conjugate, colored by atom type and shown as spheres. (b) The catalytic and suspected specificity‐determining positions of ornithine‐aminotransferase are shown as sticks and colored by conservation grade. For clarity, the backbone of the enzyme is not shown

In enzymes, mutations to catalytic residues are particularly unlikely to be tolerated, as each of these residues is engaged in a very specific function during catalysis (Figure 2). Other residues in the active site may determine the specificity of the enzyme to its cognate substrate. That is, the residues in these positions allow an enzyme to bind and act only on a certain substrate. Such positions are called specificity‐determining positions (SDPs). Notably, different forms of a given enzyme (e.g., equivalent enzymes from different species or organs) may have different residues in these positions and thus bind different substrates. Accordingly, SDPs tend to be somewhat less evolutionarily conserved than catalytic positions, which are, in essence, invariant.^46^, ^47^ Such a phenomenon can be seen in aminotransferases (also called transaminases)—a large group of enzymes that act on different substrates, such as the amino acids alanine, ornithine, aspartate, cysteine, and glutamate.^48^, ^49^ Figure 2 shows the conservation patterns of three positions in ornithine‐aminotransferase (Or‐AT), a member of the (*S*)‐selective ω‐aminotransferase enzyme family. The enzyme in this structure is bound to an inhibitor that resembles the substrate. Most of the Or‐AT positions around the inhibitor–cofactor conjugate (including the principal catalytic positions, 235 and 292) are highly conserved. Replacement of these positions by mutagenesis is likely to result in considerable loss of enzymatic activity. However, when position 85, which is also in the binding pocket of Or‐AT, is replaced, the enzyme remains active yet changes its substrate preference considerably.^50^ This suggests that position 85 is an SDP. Indeed, though position 85 is evolutionarily conserved, its conservation grade is lower than the conservation grades of the catalytic positions in the binding pocket. For example, in γ‐aminobutyrate‐aminotransferase, another member of the ω‐aminotransferase family, this position is populated by isoleucine instead of tyrosine, the equivalent residue in Or‐AT.

**Figure 3:**
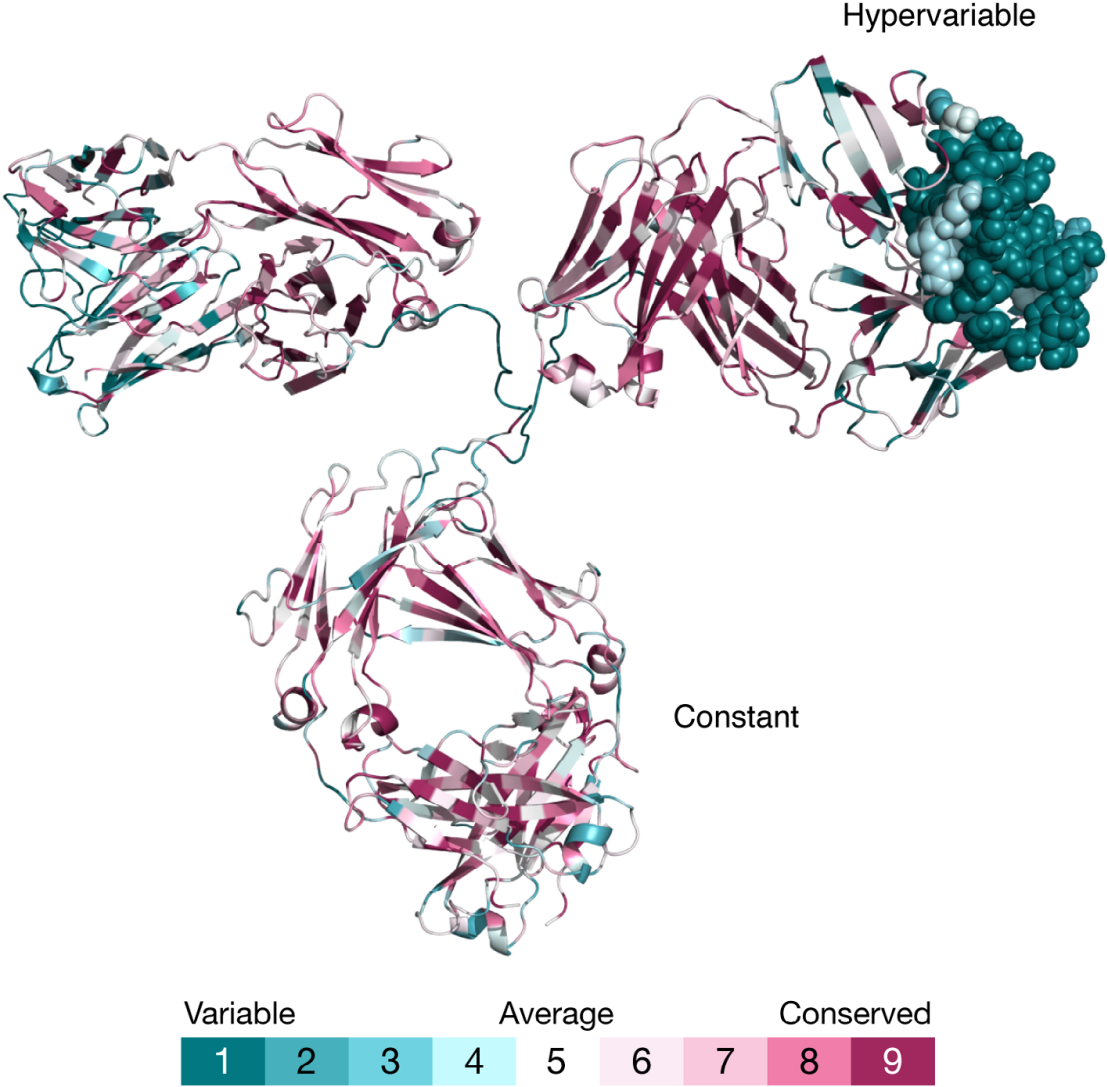
The conservation pattern of an antibody (PDB entry http://firstglance.jmol.org/fg.htm?mol=1igt). A cartoon representation of an antibody colored according to evolutionary conservation. The constant and hypervariable regions in the structure are annotated. The antigen‐binding region (CDR loops) is shown as spheres

The decreased conservation of SDPs is particularly pronounced in antibodies. This is because each antibody binds a different substrate and therefore uses different residues in the equivalent substrate‐binding positions. The SDPs in antibodies are located in the hypervariable region, at the tip of each “arm” of the antibody (Figure 3). The “stem” of this structure, referred to as the constant region, is similar in many antibodies, and it is therefore more evolutionarily conserved.

Identifying SDPs in an enzyme or antibody is not trivial and requires knowledge of the specific positions interacting with each substrate in each form of the protein. Obtaining this knowledge requires either knowing the three‐dimensional structure of the different proteins bound to their cognate substrates or biochemical data (e.g., data obtained from mutagenesis experiments) that implicate specific positions in selective substrate binding. The above examples suggest that evolutionary information, which can be obtained quickly and easily using computational tools such as ConSurf and ConSurf‐DB, not only may help researchers pinpoint functionally important positions in proteins but also may help to differentiate between subclasses of such positions (e.g., catalytic positions vs. SDPs).

## 6 CONCLUSIONS

Evolutionary information can be used to obtain valuable insights regarding the structure and function of a query protein, and in particular, it can highlight biologically important regions. ConSurf‐DB provides such evolutionary information instantly and efficiently for the majority of the proteins included in the PDB. The results are highly robust because particularly stringent thresholds were used in constructing the database. ConSurf‐DB will be periodically updated to keep up with the rapid increase in sequence and structure data.
